# Notes and Comments

**Published:** 1898-10

**Authors:** 


### Notes and Comments.[1]

Bismuth in Dental Alloy.—Mr. Thomas Fletcher suggests that a very small portion of bismuth gives a most extraordinary smoothness and plasticity to all amalgams containing silver and tin, without injuring their properties in any way. Although mixed with a small portion of mercury to pack firmly under the instrument, so perfect is its adaptation, that it can be packed against a flat surface of mother of pearl, and when hard the microscopic details of the surface which causes iridescence in the pearl are perfectly reproduced. Owing to this peculiarity it cannot be mixed or used in the hand, as it works into the pores of the skin and cannot be removed without great difficulty. In mixing the alloy, he suggests that only

[1] The assistant editor solicits contributions for this department,—new methods, new remedies and formulas, or any short practical note which may prove of value to the practitioner or student. Address 1338 Walnut Street, Philadelphia.

enough mercury should be used to give a cohesive skin to the grains, enabling them to weld together under pressure.—*The Dental Record*, London, June, 1898, p. 295.

---

Making Dental Alloy; Order in which the Metals should be added.—Mr. Thomas Fletcher considers it an error in making an alloy containing platinum to first melt this with the silver. Silver and platinum, he says, are difficult to combine, and still more difficult to keep uniform. On the contrary, platinum and tin combine energetically, with the evolution of intense heat, at a temperature below the fusing point of silver. First melt the most fusible metal, then add the others. In this manner the alloy can be made at a temperature not greatly exceeding the fusing point of the most fusible of the metals used, and overheating is entirely avoided. Want of knowledge on this point, no doubt, has caused the extraordinary statements as to the inertness of platinum in amalgam. It never got into proper combination, or was probably localized in the ingot.—*The Dental Record*, London, June, 1898, p. 295.

---

Antidote for Arsenic Erosion.—A. N. Dick, in the *Pacific Medico-Dental Gazette*, May, 1898, page 248, recommends tincture of iodine as a very reliable antidote for the erosive action produced in the soft tissues by arsenic trioxide. It has proved to be so certain and prompt in arresting the inflammation and ulceration caused by the accidental contact with the gums or cheeks that he regards it as a specific.

---

To restore Zinc for Casting.—The only way to restore zinc that has become thick by repeated heating is to heat the metal to incipient redness, throw a small quantity of strong hydrochloric acid on the surface, and stir it with a stick. About two tablespoonfuls will render a large ladleful of thick zinc perfectly fluid. This, however, does not remove the iron. That can only be separated by redistilling,—an operation impracticable on a small scale. —Mr. Thomas Fletcher in *The Dental Record*, London, June, 1898, p. 295.

Another way. An infinitesimal amount of aluminum added to thick zinc perfectly restores its fluidity. This is accomplished by first making an alloy of one part of aluminum to twenty-five of zinc. The thick zinc is heated to its fusing point, and small portions of this alloy are added until the desired effect is produced. This process is the subject of a patent. It is in commercial use, where tons of zinc, that heretofore have been practically wasted, have been restored at a mere nominal cost.

---

Death from an Abscessed Tooth.—Dr. J. H. Neeley, of Paulding, Ohio, reports in the *Ohio Dental Journal*, June, 1898, the death of a girl twelve years of age from blood-poisoning, with the following history: In September, 1896, she had a severe attack of typhoid fever, but recovered nicely. Soon after she had pain and marked swelling of the face on the right side. The lower first molar of that side, which was decayed, became loose and elongated. Fever again was present, and the physician was called, who, finding the cervical glands swollen, pronounced it a case of scrofula, and advised a course of treatment, which was granted. No attention was paid to the tooth. An abscess formed, and discharged on the face. This the physician pronounced "good," provided the discharge could be kept up, so that the other glands would not inflame and break; so for fourteen months the abscess was kept open, a compress being worn constantly to keep the saliva from flowing out. The odor was very offensive. In this condition the case came into Dr. Neeley's hands for the removal of the tooth, which by this time was a mere shell, the root being entirely absorbed. He urged the immediate removal of the diseased tissue, but was not permitted to do anything more than extract the tooth, the child being immediately taken back to her physician. She died two months later from blood-poisoning.

---

General Medical Council of Great Britain and the Dental Profession.—After many years persistent effort our professional brethren of Great Britain have won and are now represented in the General Medical Council, Mr. Charles S. Tomes having been appointed to that position for five years, filling a "crown" vacancy caused by the death of Sir Richard Quain. About the same time he was honored by being elected a Fellow of the Royal College of Surgeons, thus forestalling a possible technical objection that might have been made to his appointment. Mr. Tomes is well qualified for his unique position as the first dentist accorded membership in the General Medical Council, having been for a long time their adviser in dental matters, fulfilling in that capacity, faithfully and tactfully, many delicate commissions in a manner equally satisfactory to the council and to the dental profession. This recognition of the dental profession by electing a dentist to membership in the council will remove a just cause of dissatisfaction and lessen the friction that now and again existed between the representatives of the dental profession and the General Medical Council.—Editorial in *British Journal of Dental Science*, June 1, 1898.

